# Benthic foraminifera as bioindicators for the heavy metals in the severely polluted Hurghada Bay, Red Sea coast, Egypt

**DOI:** 10.1007/s11356-023-27242-4

**Published:** 2023-05-06

**Authors:** Ramadan M. El-Kahawy, Mohamed S. Mabrouk

**Affiliations:** Geology Department, Faculty of Science, Cairo University, Cairo, 12613 Egypt

**Keywords:** Benthic foraminifera, Bioindicators, Heavy metals, Red Sea, Hazard quotient, Hazard index

## Abstract

Twenty-nine sediment samples were collected from the Hurghada Bay, a heavily polluted bay on the Red Sea of Egypt, to inspect the environmental quality status and anthropogenic consequences on benthic foraminifera. Some foraminiferal species showed deformations in their apertures and coiling directions as a response to environmental stresses. In addition, the FoRAM index, an index used for evaluating the growth of coral reefs, indicated a hazard in the proximity of nearshore stations. To elucidate the relationships between the biological response and chemistry of sediments, eight heavy metals concentrations (Cu, Cd, Zn, Pb, As, Cr, Ni, and Mn) were analyzed using inductively coupled plasma–atomic emission spectrometers (ICP-AES). Interestingly, two groups of benthic foraminiferal associations were illustrated using multivariate statistical analyses. Group I have extremely high heavy metal concentrations, an enriched total organic matter (TOM)%, high deformation percentages, and mud content. Moreover, it is dominated by *Ammonia tepida* which is regarded as an opportunistic species. Group II includes low to moderately polluted stations, highly enriched living foraminiferal assemblages, and is dominated by the sensitive rotaliids *Neorotalia calcar* and *Amphistegina lobifera*. Alternatively, four geochemical indices, EF, CF, *I*_geo_, and PLI, are used to assess the contamination level that shown ominous spots for the nearshore stations of the Hurghada Bay. The pollution indices (HQ and HI) were also conducted to evaluate the risks of carcinogenic heavy metals on human health. Our findings demonstrated that ingestion and dermal exposure have greater carcinogenic hazards for adults and children than inhalation. The lifetime carcinogenic risk (LCR) is significantly higher than the permissible limit and follows this order: Pb > As > Cr > Cd > Ni. To that end, developing strategies to lessen the negative impact of pollution on human health and/or the Red Sea’s biodiversity is an inevitable issue in the present day and future.

**Supplementary Information:**

The online version contains supplementary material available at 10.1007/s11356-023-27242-4.

## Introduction

Human activities (domestic and industrial effluents, aquaculture, and tourism) now have a significant impact on the quality of shallow marine ecosystems in an urbanized coastal region. The coastal stretch is usually the most environmentally threatened areas, and it is an important issue to inspect the reasons of such ecological worsening. Many endeavors successfully created valuable use of micro-organisms (i.e., benthic foraminifera) as a proxy to bio-monitor changes in the coastal environments over the previous few centuries (Samir and El-Din 2001; Geslin et al. 2002; Frontalini et al. 2009, 2013; Li et al. 2021; El-Kahawy et al. 2021; Balachandar et al. 2023). For instance, certain models have been proposed scrutinizing faunal relative abundance and diversity in such environments. They are either chemicals such as heavy metals (Frontalini and Coccioni 2008; El-Kahawy et al. 2018; Price et al. 2019; Li et al. 2021; Balachandar et al. 2023) or organic matter enrichments that causes eutrophication (Alve 1991; Coccioni et al. 2009; Martínez-Colón et al. 2018).

Benthic foraminifera is a well-documenting group that regards an excellent tool to evaluate the ecosystem because they are typically abundant in coastal lagoons (Martins et al. 2015). Additionally, they are occurring and distributed throughout wide spectrum of environments as well as high species diversity and abundance which encompassing short-time span (Murray 1991). Consequently, they respond effectively quick to the short-term environmental changes either anthropogenic or natural conditions of the ecosystem; hence, this response may be displayed as shells malformations (Murray 2006; Frontalini and Coccioni 2008). The foraminiferal assemblages vary due to environmental variables, e.g., food, temperature, oxygen, pH, salinity, and substrate type (Boltovskoy and Wright 1976; Murray 2006; Frontalini et al. 2013). Interestingly, the morphological deformities (malformation) are not only constrained to the anthropogenic activities but also to natural stressed conditions such as hyper-salinity (Romano et al. 2009).

Recent benthic foraminifera on the Egyptian Red Sea coast has been the subject of several studies investigating their taxonomy, abundance, and distribution (e.g., Reiss and Hottinger 1984; Madkour 2013). Aside, several studies have been carried out on the Red Sea, particularly Hurghada area, focusing only on the geochemical indices (heavy metals) and their relations to sediment fractions (e.g., Attia and Ghrefat 2013; Nour et al. 2018). Thus, these studies lack clarity of the integration among the biological communities and enriched heavy metals in sediments. Therefore, our study adopted this approach to integrate benthic foraminifera as a pollution bio-monitor with the geochemical data.

Short-term regularly spaced bio-monitoring studies to record marine environmental changes are needed in many alarming spots along the Egyptian Red Sea coast. Hurghada site is one of the most Egyptian Red Sea marine ecosystems that has been damaged by human activities (e.g., Madkour et al. 2014). Due to decades of pollution from phosphate mining, oil exploration, sewage, and landfill leachates, hazards are now perceptible (e.g., El Metwally et al. 2017; Nour et al. 2018).

In this regard, the main goals of the present work are to as follows:

1. 1 Inspect the pollution sources and their implications on benthic foraminiferal abundance, diversity, and morphological growth
2. Assess pollution degrees using geochemical, biological, and human health risk indices
3. Evaluate the coral reefs health status using the FoRAM (Foraminifera in Reef Assessment and Monitoring) index
4. Create a comprehensive image of the potential ecological risk in the Hurghada area

## Material and methods

### Study area

**Fig. 1:**
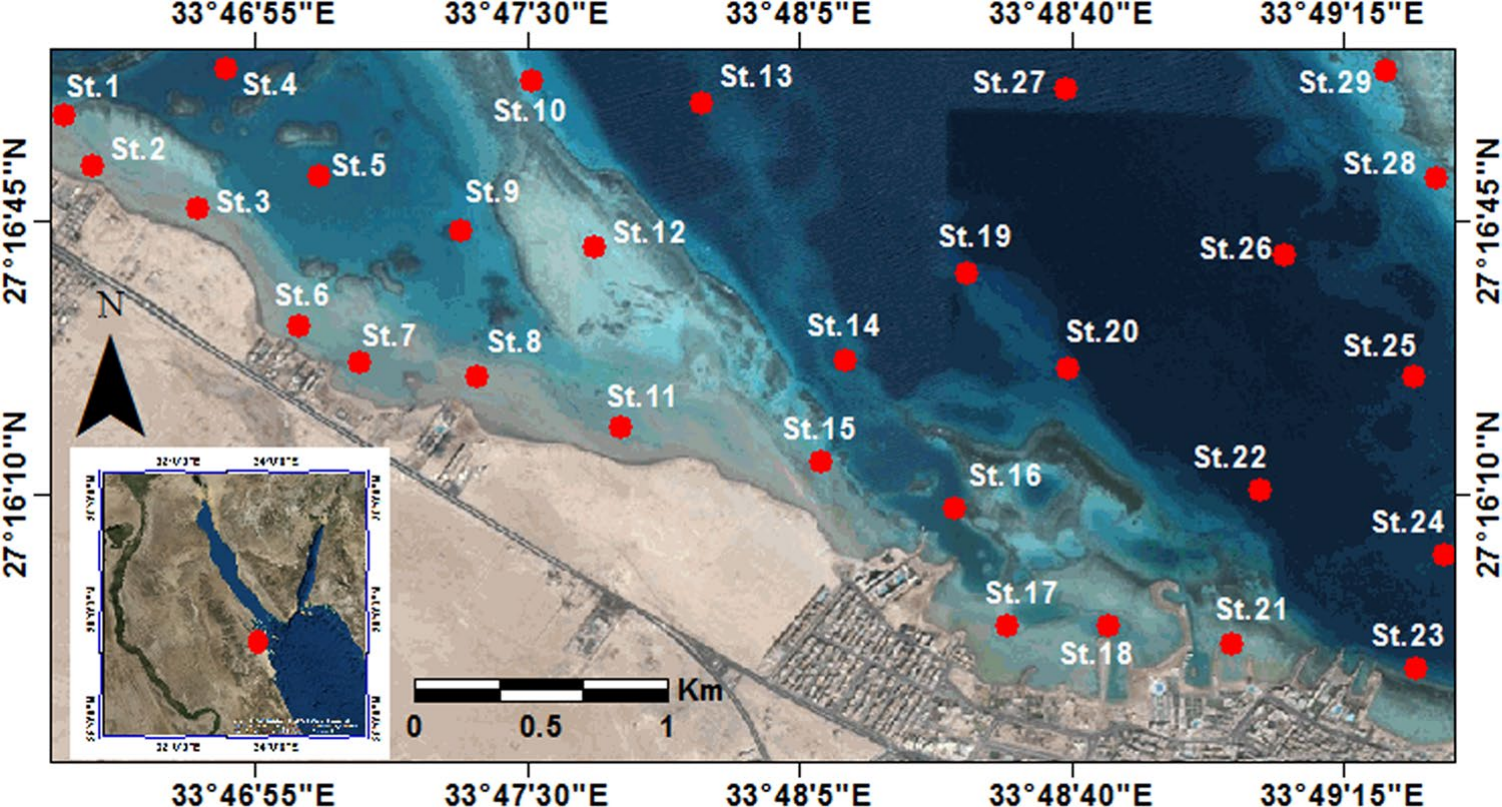
Location map for the study area shows samples distribution at the Hurghada site

**Fig. 2:**
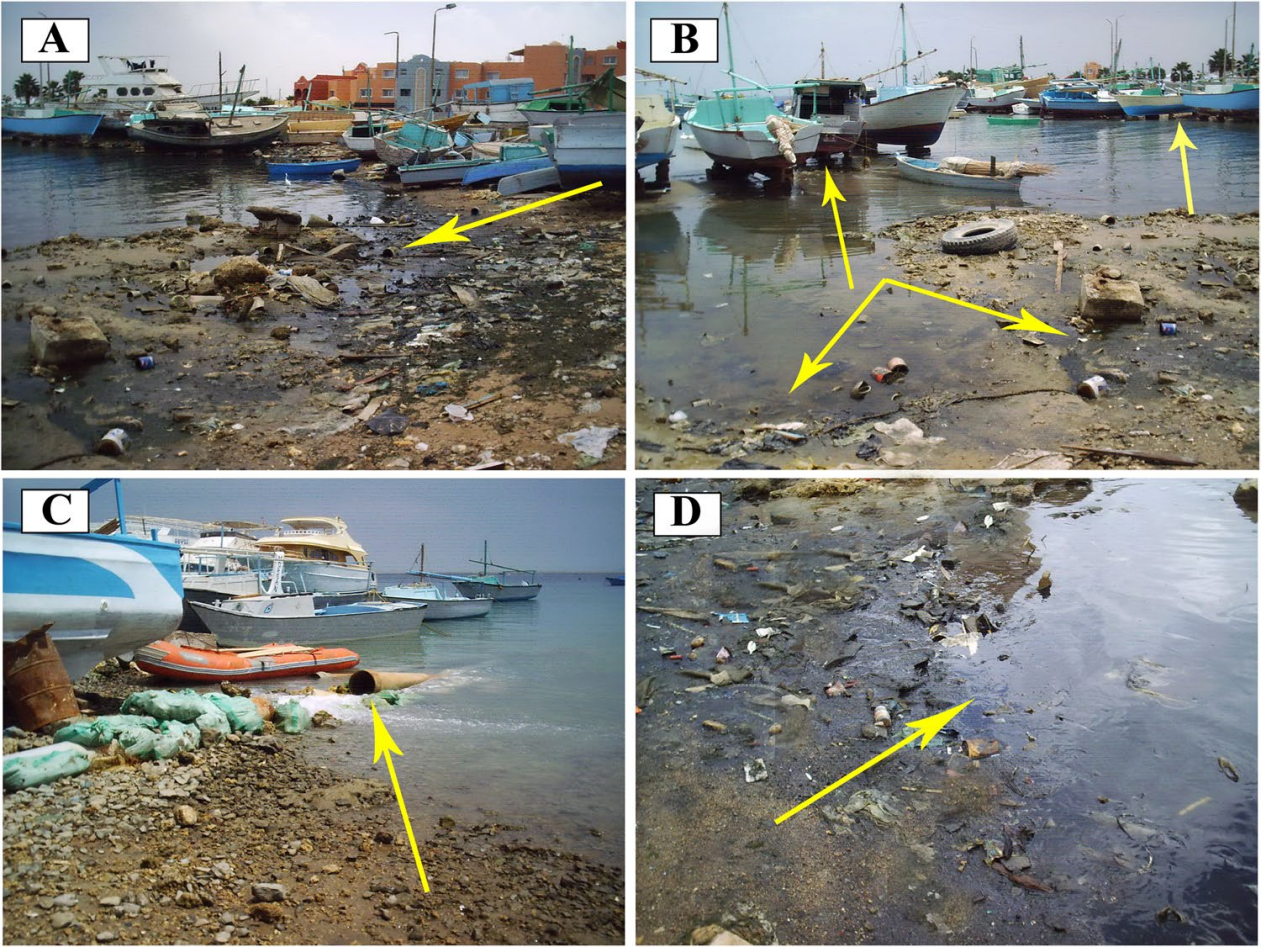
Field photos denoting anthropogenic sources of pollution in the study area: **A** and B fishing boats reclamations and garbage dumps of different waste materials directly on the coast of Hurghada site; **C** drainage of reject sewages directly effluent into the sea; **D** waste oil from the boats on the coastal part of the Hurghada Bay

The study area encompasses stations distributed along the northern Hurghada area on the Egyptian Red Sea, between lat. 27°15′ 40″ to 27°17′ 0″ N and long. 33°46′ 40″ to 33°49′ 20″ E (Fig. 1). The area of study located northerly of two main phosphate mines and their ports on the Red Sea, Umm Hawytat, and Hamrawin. Many jetties (marinas) were observed during the field survey, few for fishing boats settling, while the others for touristic and recreational use. The northern part of the study area is occupied by the main shipyard of the Hurghada (Fig. 2), whereas touristic resorts and hotels oppressed in the southern part. The beach is narrow, approximately 4 m wide, and has an inclination angle of about 3°, followed by a gently sloping muddy sand tidal flat zone. The beach is littered with solid waste. The bottom floor varies from fine sand to mud with pollutants such as plastic bags, tires, cans, and steel leftovers, then a wide back reef zone covered by biogenic sand. The area is inhabited by colonies of coral reefs, macroalgae, and mollusks. The corals were found retrograded toward sea and their densities diminished in the shallow depths. The area is dominated by small colonies of scleractinia, and octocorallia species such as *Galaxea fascicularis*, *Goniastrea retiformis*, *Acropora hyacinthus*, *Porites lutea*, and *Millepora dichotoma*. On the other hand, *Halimeda tuna* is a type of green algae was incorporated inside the branches of the coral reefs in tiny clusters. Also, seagrasses were covered the bottom of the substrate as a large patch and dominated by *Halodule uninervi*.

The Red Sea climate is influenced by the NW-NE wind direction, which is in turn orient the sea waves into NE-SW (Mansour 1995). This of course affect on the ebb and flood strength of the seawater currents. During the high tide, the water depth is reached 0.9 m while during the low tides 0.3 m. The Red Sea is generally suffering from scarce rainfall, which is represented by short duration water drops; approximately 10–15 mm/year (Morcos 1970).

### Sampling and oceanographic measurements

Twenty-nine surface samples were obtained from several environmental niches at the Hurghada site in July 2020 (Fig. 1). A plastic bottle (10 cm × 1 5 cm) was used to extract approximately 100 cm^3^ from the uppermost 0–2 cm of recent surface sediments. A Hydrolab Surveyor-4 Instrument has been used to measure the coordinates, depth, and ecological factors such as salinity, temperature, and pH (Table 1). The stations were distributed to include shallow depths (close to the hotels and tourist villages) and deeper depths (away from the coastal buildings). In water depths < 1 m, samples were collected via the plastic coring bottle, whereas the self-contained underwater breathing apparatus diving (SCUBA) method was used for collecting sediment samples > 1 m using a coring box.

**Table 1:** The water depth, physico-chemical parameters, and distribution of sand and mud percentages in the Hurghada site

Stations	Water depth (m)	Salinity‰	pH	CaCO3%	TOM%	T ^o^C	Sand%	Mud%
St.1	0.6	40.1	7.2	52.9	4.4	26.1	77.1	22.9
St.2	0.6	39.7	7.2	53.1	7.4	24.6	58.3	41.7
St.3	2.4	39.8	7.6	74.2	6.4	25.8	71.2	38.8
St.4	8.1	40.6	7.7	27.8	3.4	24.4	63.4	36.6
St.5	5.4	40.6	7.7	33.5	5.3	24.3	69.9	30.1
St.6	1.1	41.0	7.9	75.0	13.4	25.9	59.8	40.2
St.7	1.0	40.3	7.7	27.8	14.2	26.6	63.1	36.9
St.8	0.9	40.2	7.6	51.3	5.3	25.3	67.6	32.5
St.9	3.6	40.7	7.6	49.5	8.3	24.6	67.3	32.7
St.10	4.2	41.2	7.6	34.6	2.9	24.4	65.9	34.1
St.11	1.5	40.7	7.7	42.3	3.8	25.7	74.6	25.4
St.12	3.5	41.1	7.8	29.3	3.1	24.6	69.1	30.9
St.13	22.5	40.9	7.5	47.9	3.5	23.7	64.6	35.4
St.14	8.5	40.9	7.8	42.4	7.1	24.3	72.8	27.2
St.15	2.3	40.6	7.5	44.5	4.9	24.9	80.9	19.1
St.16	4.2	41.0	7.4	62.4	11.8	24.6	61.4	38.6
St.17	1.0	40.9	7.6	49.6	15.7	24.6	58.9	41.1
St.18	1.5	40.9	7.7	52.1	14.3	25.9	59.2	40.8
St.19	18.0	41.1	7.8	48.5	2.1	23.8	68.1	31.9
St.20	9.5	41.2	7.5	44.2	5.4	23.9	69.7	30.4
St.21	1.1	40.9	7.5	62.3	14.9	26.4	58.9	41.1
St.22	4.5	39.8	7.8	22.1	17.1	24.9	61.9	38.1
St.23	4.6	41.0	7.6	48.5	11.7	25.2	67.2	32.8
St.24	16.5	41.1	7.3	20.5	5.6	24.0	73.1	26.9
St.25	22.0	40.9	7.7	26.7	3.7	24.0	69.4	30.6
St.26	18.1	41.2	7.6	53.1	9.2	24.1	67.1	32.9
St.27	29.4	40.8	7.5	41.3	2.9	23.5	63.8	36.2
St.28	7.6	41.6	7.7	29.5	13.3	24.0	68.8	31.2
St.29	2.3	41.1	7.4	56.2	14.3	24.5	65.1	34.9

### Foraminiferal analyses

The foraminiferal analysis follows the foraminiferal bio-monitoring procedures developed by Schönfeld et al. (2012). Rose Bengal dye with 70% ethanol solution (2 g/l) was used for staining the cytoplasm to differentiate living from dead organisms.

Approximately 50 g of each sample was treated using 5% hydrogen peroxide to disintegrate the organic matter and washed over a 63-µm sieve to eliminate the finest fractions. The residue was dried at 80 °C and utilized for the foraminiferal examination. The foraminiferal assemblages were also inspected using a binuclear Leica microscope and classified based on the generic identification of Loeblich and Tappan (1988). According to Schönfeld et al. (2012), benthic foraminiferal assemblages > 125 µm were examined and identified, whereas those smaller than this size are neglected. The living and dead specimens as well, have been counted for each sample. Accordingly, the diversity indices were used to assess the interrelationships between abundance and species richness; Fisher alpha index (Fisher et al. 1943) and dominance index (D). The Paleontological Statistics Program, version 3.17, has been used to quantify these indices (Hammer et al. 2009).

Since the Red Sea is a favorable ecological niche for growth of coral reef, it is vital and significant to assess the health of coral reefs communities in the area of study. Thus, the Foraminifera in Reef Assessment and Monitoring index (FoRAM Index; FI) is calculated as adopted by Hallock et al. (2003). Also, we estimated the foraminiferal abnormality index (FAI), the proportion of deformed specimens in each sample, and the foraminiferal monitoring index (FMI), which is the percentage of malformed specimens in each species of the assemblage (Coccioni et al. 2005). At the Egyptian Mineral Resources Authority (EMRA), the distorted specimens were scanned using a scanning electron microscope.

### Grain size analyses

Granulometric analysis was conducted on around 30-g sediments. Each sample was treated with dilute HCl and 15% H_2_O_2_ to remove the carbonate and organic matter respectively. The wet fractions sieved over five different sieving meshes (500 µm, 350 µm, 250 µm, 125 µm, and 63 µm). The proportion (wt.%) of each sieve was weighed after they oven dried at 50 °C, then the sediments classified following the method described by Folk and Ward (1957). The organic matter content was determined using 2 g of sediments via sequential weight loss at 550 °C. The samples were weighed again without organic matter to quantify the weight proportion following the method adopted by Dean (1974).

An acid treatment weight loss procedure has been used to estimate the carbonate content (Gross 1971). The residue is collected on a 0.45-m filter after being treated with a diluted HCl acid solution (2.5 N). Drying the filter led to a measured weight loss, which was then converted to a percent of carbonate (Table 1).

### Geochemical analyses of sediments

The concentrations of eight heavy metals for 29 sediment samples were valued in the EMRA laboratory using ICP-AES. The sediment samples (63 µm) were rinsed with sodium hypochlorite for 24 h before being soaked with distilled water. At 60 °C, the samples were dehydrated and pulverized into powder. In a 3 ml HClO_4_ + 5 ml HNO_3_ + 15 ml HF solution, 0.2 g of the prepared sediment sample has been processed. The sample was poured into a 100-ml flask once the 50 ml of HCl (1:1) was injected. A calibration curve was drawn using a series of variable standards. Then, the true sample concentration monitored via the Agilent-720 ICP-AES equipment. The cutoff levels for detecting these eight heavy metals are as follows: Cu (1 mg/kg), Zn (1 mg/kg), Mn (2 mg/kg), Cd (0.2 mg/kg), As (2 mg/kg), Pb (3 mg/kg), Cr (1 mg/kg), and Ni (1 mg/kg) (Table 2).

**Table 2:** Heavy metal concentrations (mg/kg) in the bottom sediments of the Hurghada site

Stations	Cu	Pb	Zn	Cd	Ni	Mn	Cr	As	Fe
St.1	15.1	17.5	29.5	2.6	35.7	172.3	87.0	11.4	1500
St.2	22.1	21.3	14.2	3.1	35.6	174.8	60.0	23.6	5800
St.3	14.5	8.3	13.7	1.9	35.7	50.6	40.0	2.0	3800
St.4	11.3	9.8	5.2	0.5	21.1	16.4	19.7	6.8	1600
St.5	23.2	27.3	10.4	0.7	42.9	86.0	21.7	6.1	3700
St.6	14.6	63.2	26.2	3.2	49.8	26.2	71.5	21.4	2100
St.7	23.1	55.3	25.0	3.7	14.3	15.5	42.8	19.4	2100
St.8	16.3	36.5	23.4	1.7	28.1	231.5	78.4	17.2	2800
St.9	16.5	29.4	11.4	1.8	63.8	22.9	19.5	7.9	5600
St.10	18.5	10.4	5.7	0.4	35.6	27.5	9.8	3.9	2900
St.11	19.1	13.6	12.4	3.5	28.5	92.1	39.8	9.2	7500
St.12	15.8	11.3	7.3	1.1	49.9	266.0	19.7	6.3	5400
St.13	5.8	35.2	15.3	0.9	25.2	22.5	6.4	5.3	4400
St.14	11.9	42.3	23.6	1.7	17.0	62.8	18.0	11.2	7800
St.15	19.6	34.6	17.5	1.7	28.6	26.1	36.7	13.2	7900
St.16	18.1	79.4	27.4	3.1	70.1	245.3	90.3	24.4	8700
St.17	25.1	89.4	34.2	4.6	85.7	272.6	80.8	22.3	8900
St.18	24.1	84.1	32.4	3.8	67.8	137.5	62.8	19.8	9900
St.19	8.1	37.2	14.6	0.9	27.0	86.1	11.2	4.3	4800
St.20	6.8	34.5	11.8	1.1	7.1	54.7	11.2	7.8	6200
St.21	21.5	73.1	30.6	3.7	63.4	126.5	49.8	20.6	11,300
St.22	15.7	61.4	21.4	2.9	85.7	153.2	39.6	14.8	8700
St.23	19.6	51.4	22.3	2.6	36.8	74.9	37.4	15.9	10,200
St.24	11.2	39.1	14.2	1.2	21.2	50.1	15.3	9.3	5900
St.25	12.0	37.1	15.4	1.2	21.5	85.5	16.0	5.8	5500
St.26	10.7	43.1	7.6	2.3	17.1	146.7	21.2	10.6	4900
St.27	7.1	21.3	3.8	0.5	31.7	52.1	8.0	3.1	3400
St.28	12.8	9.7	8.7	1.9	33.2	55.0	7.5	3.9	4500
St.29	7.4	36.7	25.3	0.7	20.6	51.5	17.7	7.8	4100
Background:(Hanna 1992)	4.09	19.5	9.05	0.3	8.86	112	–––-	–––-	–––-
Mansour et al. (2011)	4.1	19.5	9.1	0.3	8.9	112	–––-	–––-	–––-
Attia & Ghrefat (2013)	13.14	43.56	15.76	3.11	33.67	77	–––-	–––-	–––-
Nour (2018)	1.26	42.38	7.77	0.14	1.74	51.95	–––-	–––-	–––-
Mean (present study)	15.4	38.4	17.6	2	38	99.5	35.9	11.6	–––-

The interrelationships among the diversity, total organic matter (TOM%), and heavy metal toxicity were correlated via Pearson’s correlation coefficients (*p* < 0.01) (Table 3).

**Table 3:** Correlation matrix showing the relationship among diversity, organic matter, and heavy metals

	*Diversity*	*TOM%*	*Cu*	*Pb*	*Zn*	*Cd*	*Ni*	*Mn*	*Cr*	*As*
Diversity	1									
TOM%	− 0.73	1								
Cu	− 0.82	0.54	1							
Pb	− 0.94	0.78	0.85	1						
Zn	− 0.90	0.75	0.84	0.95	1					
Cd	− 0.91	0.67	0.9	0.96	0.95	1				
Ni	− 0.91	0.71	0.87	0.98	0.92	0.94	1			
Mn	− 0.85	0.64	0.76	0.91	0.85	0.88	0.9	1		
Cr	− 0.92	0.65	0.89	0.94	0.95	96	0.92	0.87	1	
As	− 0.91	0.71	0.89	0.95	0.96	0.96	0.93	0.84	0.96	1

### Assessment of the metal pollution

Four geochemical indices, enrichment factor (EF), contamination factor (CF), geo-accumulation index (*I*_geo_), and pollution load index (PLI), were used to assess the heavy metals contamination levels in the Hurghada Bay. Firstly, opting a heavy metals background for geochemical investigations is the base for an environmental evaluation. Natural air deposition of metals and weathering of bedrock are responsible for regulating background levels of heavy metals. Many authors have utilized Turekian and Wedepohl (1961) average shale concentrations as a reference baseline (Singh et al. 2005; Pekey 2006; Varol 2011). Aside, other authors have established that local background values provide more reliable findings than global background values triggered by sediments textures differences from place to place (Rubio et al. 2000; Sakan et al. 2009). Therefore, the data of Hanna (1992) exploited to obtain the background values. On one hand, Summers et al. (1996) proposed a straightforward strategy for distinguishing between natural and human impacts. To evaluate whether the sediment sample was metal enriched compared to a control sample, the metal concentrations were normalized to iron.

The EF is regarded as a powerful technique for assessing the degree of natural or anthropogenic sediment contamination (Chen et al. 2007). The EF categorized into seven classes as shown in Table (4a), and was computed using the following equation:

$$\mathrm{EF}=\frac{\left(\frac{\mathrm{metal}}{\mathrm{Fe}}\right)\mathrm{sample}}{\left(\frac{\mathrm{metal}}{\mathrm{Fe}}\right)\mathrm{background}}$$

The geo-accumulation index (*I*_geo_) was established by Müller (1979) to assess the degree of pollution in sediments by comparing the current condition with pre-industrial levels. Seven classes have been discriminated by Müller (1981) as illustrated in Table (4b) using the following equation:

$$I\mathrm{geo}=\frac{{\mathrm{log}}_{2}(Cn)}{1.5(Bn)}$$

where Cn represents the current metal concentration, Bn is the geochemical background value of the same metal, and factor 1.5 is the correction factor of the background.

The CF is the ratio obtained by dividing the concentration of each metal analyzed in the sediment to the background value as suggested by Hakanson (1980). Four classes were categorized and illustrated in Table (4c).

$$\mathrm{CF}=\frac{\mathrm C\;\mathrm{heavy}\;\mathrm{metal}}{\mathrm C\;\mathrm{background}}$$

PLI is a method widely applied to address the prevalent consequence of metal contamination. PLI is obtained from the following formula:

$$\mathrm{PLI}={\left({\mathrm{CF}}_{1}\times {\mathrm{CF}}_{2}\times {\mathrm{CF}}_{3}\times {\mathrm{CF}}_{4}\times \dots \dots .\times {\mathrm{CF}}_{\mathrm{n}}\right)}^{1/\mathrm{n}}$$

where *n* is the number of measured metals. The pollution level deduced as PLI > 1 means pollution exists, while PLI < 1 has no pollution (Table 5a).

#### Sediment quality guidelines

The sediment quality guidelines (SQGs) are used to create monitoring programs to evaluate the possible ecological threats caused by dredged materials. Additionally, SQGs are exploited to detect contaminants in aquatic habitats (Persuad et al. 1993; Long and MacDonals 1998). The impacts on sediment-dwelling organisms due to heavy metals were investigated by comparing the measured concentrations of heavy metals with the SQGs reported by Persuad et al (1993). Accordingly, two levels of risk were considered; firstly, the lowest effect level (LEL), where sediments are clean/pristine, and there is no negative impact on marine biota when measured values are at or below this level. The second, is the severe effect level (SEL), which indicates that organisms living in the sediments will be negatively impacted by the pollution (Table 5a).

#### Risk assessment of human health

The health-risk assessment indices were calculated using the equations developed by USEPA (2002); USEPA (2011); Luo et al. (2012); Aendo et al. (2022). The average daily intake (ADI) of heavy metals in the sediments of the present study is used to perform an exposure assessment, and thereby determine the hazards to human health. The ADI was investigated using three paths: ingestion (oral), inhalation, and dermal contacts for both children and adult.

## Non-carcinogenic risk assessment

The non-carcinogenic heavy metals were evaluated based on hazard quotient (HQ) and hazard index (HI). The HQ is a measure of the non-carcinogenic, and health risks of heavy metals in sediments triggering chronic and non-carcinogenic effects. It is calculated based on the ADI of each element and the reference dose (RfD). The HQ was calculated using the equation below (Weissmannová and Pavlovský 2017):

$$\mathrm{HQ}=\frac{\mathrm{ADI}}{\mathrm{RfD}}$$

The hazard index (HI) is expressed by summation of HQ for each metal as described via equation below (USEPA 2009).

$$\mathrm{HI}=\sum \mathrm{HQ}$$

When HQ or HI $\le$ 1, this indicates there is no evidence of a health risk from exposure to non-carcinogenic metals., while HQ or HI > 1 there may be potential non-carcinogenic effects on human health (USEPA 1999, 2011).

## Carcinogenic risk assessment

Carcinogenic risk (CR) was calculated throughout the incremental probability of acquiring cancer throughout a lifetime as a result of exposure to a potential carcinogen (USEPA 2011) as follows:

$$\mathrm{CR}=\mathrm{ADI}\times \mathrm{SF}$$

where SF is cancer slope factor (mg/kg/day) through the three paths (see appendix 2).

$$\mathrm{LCR}=\sum \mathrm{CR}$$

If the value of lifetime carcinogenic risk (LCR) exceeds 1 × 10^−4^, it means a lifetime risk on the human body (USEPA 1989, 2011).

### Statistical analyses

The similarity and dissimilarity of the samples and species were measured via heatmap hierarchical cluster analyses (HCA). Both Q- and R-modes were executed using XLSTAT software to construct dendrograms representing the samples and species associations. For only the statistical analysis, living benthic foraminiferal individuals were used rather than dead organism, to evaluate the ecological quality status (Schönfeld et al. 2012). We relied on the living benthic foraminifera individuals to avoid the biased that could be resulted from taphonomic processes (e.g., transportation, and destruction). Accordingly, approximately 300 adult living individuals were retrieved from each sample to perform effective statistical treatment methods. The samples of low benthic foraminiferal abundances were normalized before statistical analysis to lessen the environmental variables effects on the faunal distribution. Species with occurrences greater than 3% in at least one sample were used in the cluster analysis. Using Ward method and squared Euclidean distance, the species and samples were discriminated forming dendrograms.

To visualize the relationship among the environmental variables and the faunal gradients, multivariate analyses were performed. Using CANOCO software version 5.12, detrended correspondence analysis (DCA) has been applied on the dominant living taxa (more than 3% relative abundance), to choose either applying unimodal method (canonical correspondences analysis; CCA) or linear method (redundancy analysis; RDA). The length of the first gradient is the main controlling factor in the decision regarding the preferred ordination type. According to Šmilauer and Lepš (2014), if the gradient length is shorter than 3.0 standard deviations (SDs), the linear method is recommended, which comprises constrained (RDA) and unconstrained (principal correspondence analysis; PCA) techniques. The first gradient of the detrended correspondence analysis has a length of 2.0 standard deviations, suggesting a linear method (i.e., RDA) as recommended by Šmilauer and Lepš (2014). Accordingly, RDA is used to analyze the data set to assess the ecological relationships between the measured station variables and their faunal associations. The data is transformed logarithmically and standardized by means of species centering. The RDA was performed via Monte-Carlo permutation with 499 iterations under the reduced model.

## Results

### Oceanographic data

The surface water temperature of the Red Sea at Hurghada ranges between 23.5 and 26.6 °C, with higher temperatures at the coastal stations than at deeper ones. The lowest temperature was measured at the station St.27 (23.5 °C), whereas the highest temperature was recorded at St.7 (26.6 °C) (Table 1).

The salinity fluctuated between 39.7 to 41.6 practical salinity units (PSU). The lowest salinity value was observed at St.2 (39.8 PSU), whereas the highest was at St.28 (⁓41.6 PSU). This clarifies that the Red Sea water around the Hurghada area is hypersaline.

The pH of the seawater ranges between 7.2 and 7.9, where the lowest is observed at St.1, while the highest is at St.6 (Table 1). The sediments are enriched in carbonate content; the lowest percentage was recorded at St.24 (20.5%), while the highest percentage was detected at St.6 (75.5%). The overall pattern of carbonate distribution trends is of high percentages in the northern and southern sectors (Table 1).

### Bottom sediment characteristics

#### Grain size distribution

**Fig. 3:**
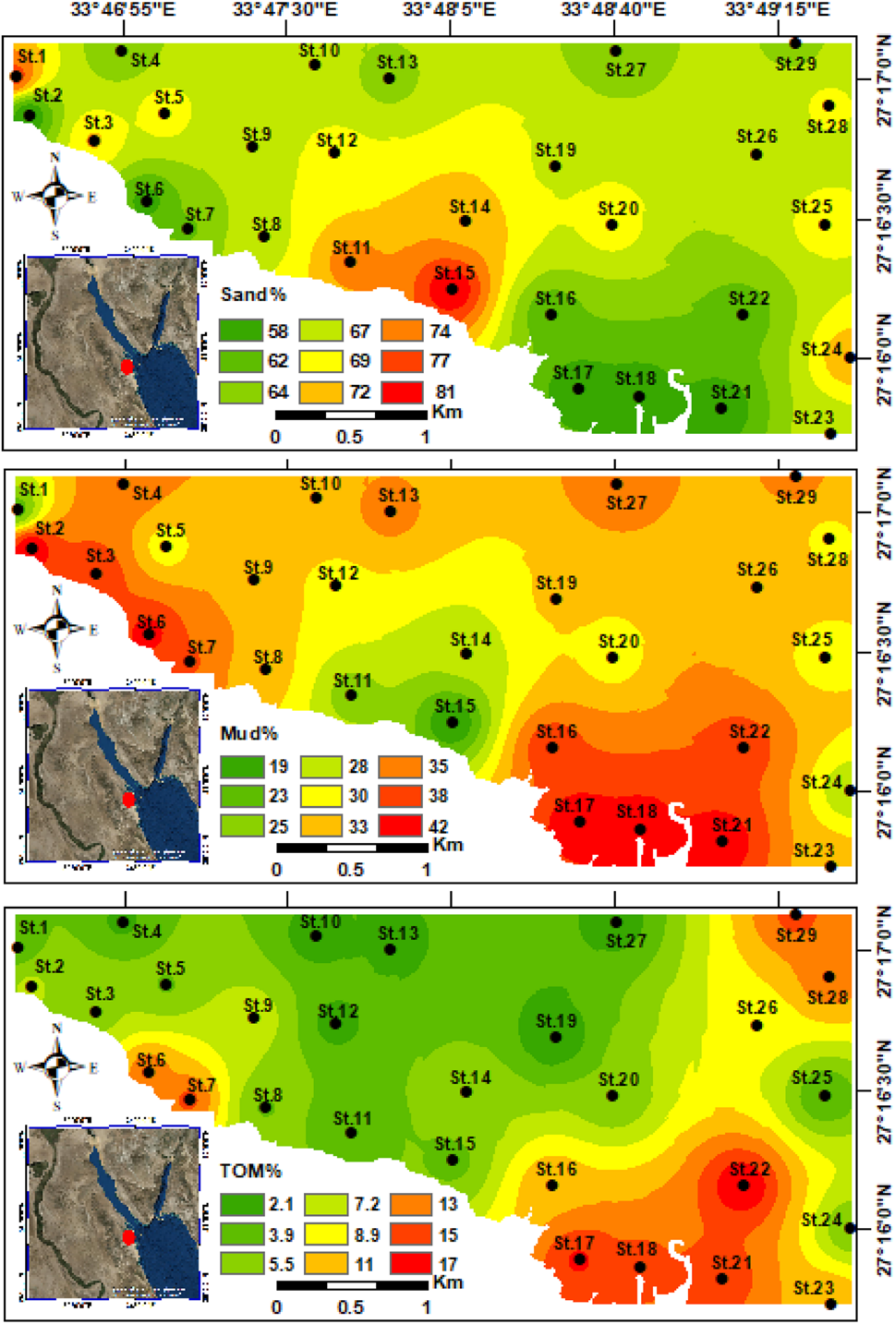
Distribution maps for the bottom facies (sand and mud %) and total organic matter in the study area

The sand fraction (> 50%) was the most constituent, whereas the mud > 35%. The mud fraction is higher in the coastal nearshore area of the Hurghada area, especially in front of the tourist buildings and hotels (Fig. 3). The central part of the area contains the highest sand fraction, which reaches up to 75%. The northwestern and southeastern sectors encompass the highest mud content (> 40%) (Fig. 3). The sample containing the highest sand fraction (80.9%) was collected from St.15, whereas the highest mud fractions (41.1%) were compiled from St.2 and St.17 (Fig. 3).

#### Total organic matter content (TOM)

The organic matter content is extremely high at the area of study; however, it fluctuates throughout all stations. The stations located within central part exhibits low values compared to the southeastern and northwestern parts of the Hurghada site. The TOM values ranged from 2.1 to 15.7%. The highest values were observed in the southeastern and northwestern sectors, where the lowest value had been detected at St.19, while St.17 has the highest TOM% and averaged 7.6% (Fig. 3).

#### Metals concentrations, contamination levels, and sediment quality guidelines

**Fig. 4:**
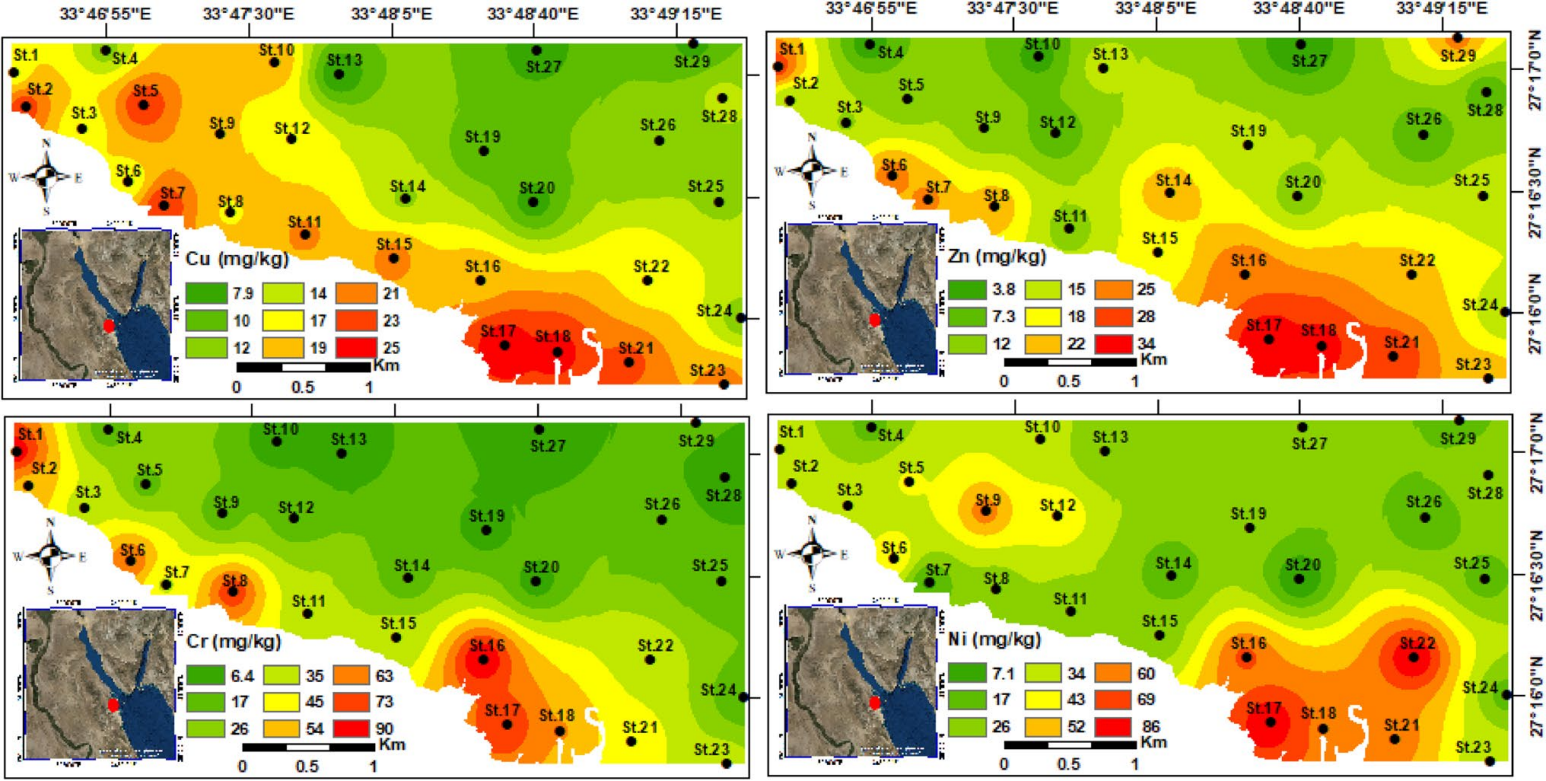
Spatial distribution maps for the heavy metals (Cu, Zn, Cr, and Ni) concentrations in the Hurghada site

**Fig. 5:**
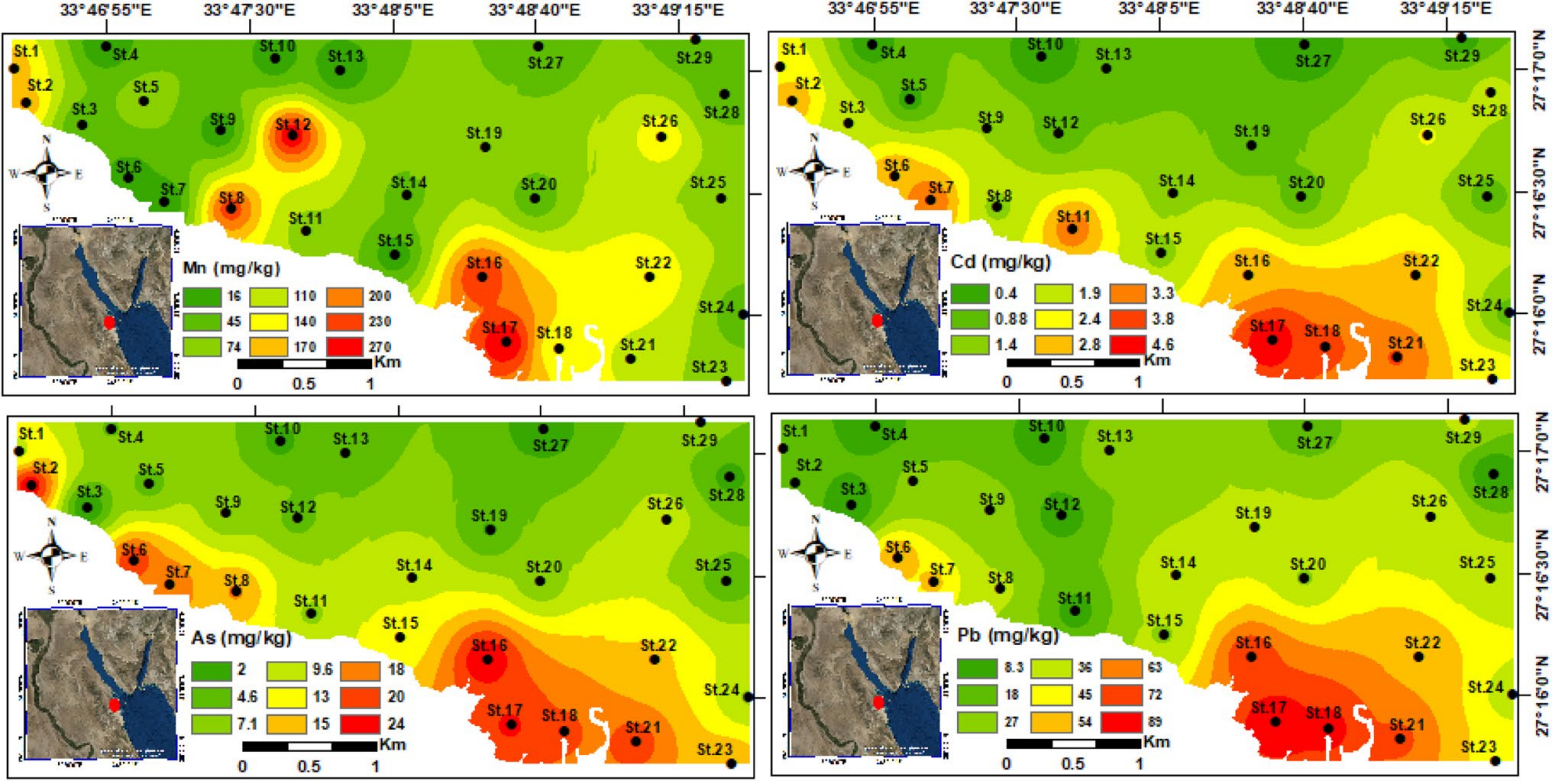
Spatial distribution maps for the heavy metals (Mn, Cd, As, and Pb) concentrations in the Hurghada site

The concentrations of heavy metals are summarized in Table 1, and spatially illustrated on geochemical maps to show their distributional patterns (Figs. 4 and 5). The highest heavy metal concentrations were observed in nearshore stations of the coastal area for the southeastern and northwestern parts, while the lower concentrations detected towards the open marine. The highest concentrations of six heavy metals (Cu, Zn, Ni, Mn, Pb, Cd) were measured in the southeastern stations at St.17, whereas St.16 exhibited the highest concentrations of Cr and As (Figs. 4 and 5).

Correlation matrix was accomplished for the heavy metals, TOM% and benthic foraminiferal diversity at the Hurghada stations, showing high positive correlation coefficients among elements (Table 3). The species diversity displayed very high negative correlations with all heavy metals and TOM%. Furthermore, the TOM% exhibited strong positive correlation coefficients with the correlated metals except Cu, and Mn, which display moderate correlation (Cu = 0.54, *p* < 0.01), and (Mn = 0.64, *p* < 0.01) (Table 3).

The calculated values of EF exhibited a significant enrichment sequence; Pb ≥ Cd ≥ Ni > Zn > Mn > Cu (Table 4a). It illustrates a moderate to severe enrichment for Pb (1.8–30.1), a minor to severe enrichment for Cd (1.1–13.2), no to moderate enrichment for Ni (0.21–4.5), no to minor enrichment for both Zn (0.14–2.5), Mn (0.11–3), and Cu (0.19–1.7). Moreover, the *I*_geo_ values (Table 4b) of this study classified the marine sediments as uncontaminated by Cu and Zn, uncontaminated to moderately contaminated by Mn and Ni, moderately to strongly contaminated by Pb and Cd. The contamination factor (Table 4c) revealed heavy metals sequence as: Pb > Cd > Ni > Cu ≥ Zn ≥ Mn in all stations. The minimum and maximum calculated CF values of the Pb have been recorded at St.3 = 2.8 and St.17 = 29.8, respectively, which is regarded as a significant indicator of pollution, followed by Cd at St.10 = 1; St.17 = 11.5, with the same behavior in most stations of the Hurghada site. Aside, Ni (St.20 = 0.44; St.17 = 5.36) shows a moderate to considerable pollution, while Cu (St.13 = 0.3; St.17 = 1.43), Zn (St.27 = 0.16; St.17 = 1.43), and Mn (St.7 = 0.13; St.17 = 2.35) recorded the least pollution levels. Furthermore, the calculated values of PLI (Table 5a) ranged between 0.69 and 4.54. The PLI values suggest only three unpolluted stations (St.4, St.10, and St.27) with PLI less than 1, while the other stations are higher than 1. The highest calculated values of PLI are distributed along the southeastern stations St.17, St.18, St.16, St.21, and St.23 (Table 5a).

**Table 4:** The calculated geochemical indices of the Hurghada site.

a		
Metal	Mean	Enrichment factor
Cu	0.61	No enrichment
Pb	7.94	Moderately severe enrichment
Zn	0.51	No enrichment
Cd	3.4	Moderate enrichment
Ni	1.53	Minor enrichment
Mn	0.55	No enrichment
< 1 = no enrichment,	3–5 = moderate enrichment,	10–25 = severe enrichment,
< 3 = minor enrichment,	5–10 = m. severe,	25–50 = very severe enrichment,
> 50 = exteremely severe enrichment		
b		
Metal	Mean	Geo-accumulation index (Igeo)
Cu	− 0.88	Low contamination
Pb	2.8	Moderately to strongly contaminated
Zn	− 1.24	Uncontaminated sediments
Cd	1.47	Moderately contaminated
Ni	0.45	Uncontaminated to moderately contaminated
Mn	− 1.28	Low contamination
Class 0 (*I*_geo_ ≤ 0) = uncontaminated(UC),		Class 3(2 < *I*_geo_ ≤ 3) = MC to strongly contaminated (SC),
Class 1(0 < *I*_geo_ ≤ 1) = UC to moderat contaminated (MC),		Class 4(3 < *I*_geo_ ≤ 4) = SC,
Class 2(1 < *I*_geo_ ≤ 2) = MC,		Class 5(4 < *I*_geo_ ≤ 5) = SC to exteremly contaminated (EC),
Class 6(*I*_geo_ ≥ 5) = EC		
c		
Metal	Mean	Contamination level
Cu	0.88	Low contamination
Pb	12.8	Very high contamination
Zn	0.73	Low contamination
Cd	5.09	Considerable contamination
Ni	2.37	Moderate contamination
Mn	0.86	Low contamination
	CF < 1 = low contamination	3 < CF < 6 = considerable contamination
	1 < CF < 3 = moderate contamination	CF > 6 = extremely high contamination

^a^Enrichment factor, ^b^geo-accumulation index, and ^c^contamination factor of the Hurghada site

**Table 5:** a- The calculated pollution load index (PLI) of the Hurghada stations, and b- comparison of the mean heavy metals of the present work with the sediment quality guidelines (SQGs)

a									
Stations	PLI	Status	Stations	PLI	Status	Stations	PLI	Status	
St.1	2.26	Polluted	St.11	1.78	Polluted	St.21	3.39	Polluted	
St.2	2.27	Polluted	St.12	1.65	Polluted	St.22	3.07	Polluted	
St.3	1.35	Polluted	St.13	1.09	Polluted	St.23	2.36	Polluted	
St.4	0.69	Unpolluted	St.14	1.69	Polluted	St.24	1.43	Polluted	
St.5	1.62	Polluted	St.15	1.59	Polluted	St.25	1.59	Polluted	
St.6	2.18	Polluted	St.16	3.61	Polluted	St.26	1.67	Polluted	
St.7	1.74	Polluted	St.17	4.54	Polluted	St.27	0.89	Unpolluted	
St.8	2.34	Polluted	St.18	3.68	Polluted	St.28	1.26	Polluted	
St.9	1.58	Polluted	St.19	1.46	Polluted	St.29	1.33	Polluted	
St.10	0.88	Unpolluted	St.20	1.04	Polluted				
b									
Parameter	Cu	Pb	Zn	Cd	Ni	Mn	Cr	As	Fe
Mean	15.4	38.4	17.6	2	38	99.5	35.9	11.6	5582.8
Minimum	5.8	8.3	3.8	0.4	7.1	15.5	6.4	2	1500
Maximum	25.1	89.4	34.2	4.6	85.7	272.6	90.3	24.4	11,300
LEL (Persuad et al. 1993)	16	31	120	0.6	16	460	26	6	20,000
SEL (Persuad et al. 1993)	110	250	820	10	75	1100	110	33	40,000

Regarding the SQGs, the average concentrations of Zn (17.60 mg/kg), Cu (15.43 mg/kg), and Mn (99.48 mg/kg) are lower than both the LEL and SEL values (Table 5b). On the other hand, the mean concentrations of Cd, Pb, Ni, As, and Cr (2.03 mg/kg, 38.40 mg/kg, 37.96 mg/kg, 11.56 mg/kg, and 35.86 mg/kg, respectively) are higher than the LEL and lower than the SEL level (Table 5b).

### Risk assessment of human health

The noncarcinogenic hazard indices HQ and HI (Table 6a) for both children and adults clarify that children are higher than adults through the three paths (ingestion, inhalation, and dermal contact) of exposure. The HI for children exhibited a decreasing sequence as follows; As > Pb = Cr > Mn > Cd > Ni > Cu > Zn, while for adult shows As > Pb > Mn > Cr > Cd > Ni > Cu > Zn. However, the HQ and HI values for children and adults are less than 1.

**Table 6:**
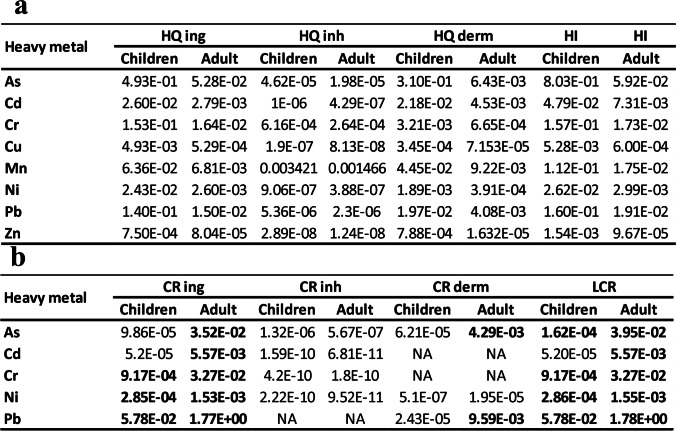
a- The calculated hazard quotient due to heavy metal ingestion, inhalation, and dermal effect and hazard index of children and adult, b- The carcinogenic risks of the analyzed heavy metals via ingestion, inhalation, and dermal for the adults and children and their lifetime carcinogenic risk (LCR)

The carcinogenic risk (CR) of children and adults displayed a significant decreasing order for ingestion path; Pb > Cr > Ni > 10E-04, and Pb > As > Cr > Cd > Ni > 10E-04, respectively (Table 6b). The LCR of Pb, As, Cr, Ni, and Cd are higher for adults than for children, with maximum LCR of Pb for children is 5.78E-02, and 1.78E + 00 for adults. Also, the dermal contact showed a risk for adults higher than children, especially the Pb (9.59E-03), and As (4.29E-03), respectively.

### Benthic foraminiferal distribution

The investigated samples yielded 34 total benthic foraminiferal species (living and dead). They are classified into twenty-one genera belonging to three suborders Textularina, Rotaliina, and Miliolina (Appendix 1). *Quinqueloculina*, *Elphidium*, *Peneroplis*, *Amphistegina*, *Ammonia*, and *Sorites* were the six main constituting genera at 17.2%, 16%, 10.2%, 10%, 9.8%, and 6.8%, respectively. Other frequent genera are as follows: *Triloculina* (5.5%), *Neorotalia* (5.4%), *Coscinospira* (4.4%), and *Rosalina* (3.7%).

The suborder Rotaliina has been the most vital part of the reported foraminiferal assemblages constituting 54% of the total foraminiferal assemblages from this study (Appendix 1). *Ammonia beccarii* (6.7%) is the most widely distributed species, followed by *Elphidium striatopunctatum* 5.8%, *Elphidium crispum* 5.6%, *Neorotalia calcar* 5.5%, *Amphistegina lessonii* 5.1%, and *Amphistegina lobifera* 4.8%.

**Fig. 6:**
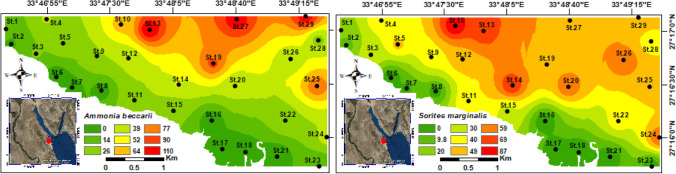
Distribution maps for the relative abundance of the two most abundant representative taxa of suborder Rotaliina and Miliolina

*A. beccarii* is the most abundant species in all stations. Figure 6 shows the *A. beccarii* peaks in the offshore stations, such as St.29, St.27, and St.13, whereas the southeastern and northwestern stations closer to the coastline, exhibit the lowest abundance around St.16, St.17, St.18, St.21, St.23, St.6, St.7, St.8, and St.7. The second most dominant species is *E. striatopunctatum.* It fluctuates between 5 and 6% in the nearshore stations, and the highest abundance was recorded in the offshore stations, especially at St.25, St.27, and St.29 (Appendix 1).

The suborder Miliolina displays a high relative abundance, representing 45.4% of the total foraminiferal assemblages (Appendix 1). *Sorites marginalis* is the most predominant species in this suborder, constituting 6.7% of the total foraminiferal association. *Sorites* shows variability across all the stations. It exhibits high abundance values in the offshore stations, whereas it reaches the lowest abundance values at St.6, St.16, St.17, and St.18 (Fig. 6). *Peneroplis platanus* (5.5%), *Peneroplis pertusus* (4.8%), and *Quinqueloculina seminulum* (4.7%) are the other prominent species (Appendix 1).

**Fig. 7:**
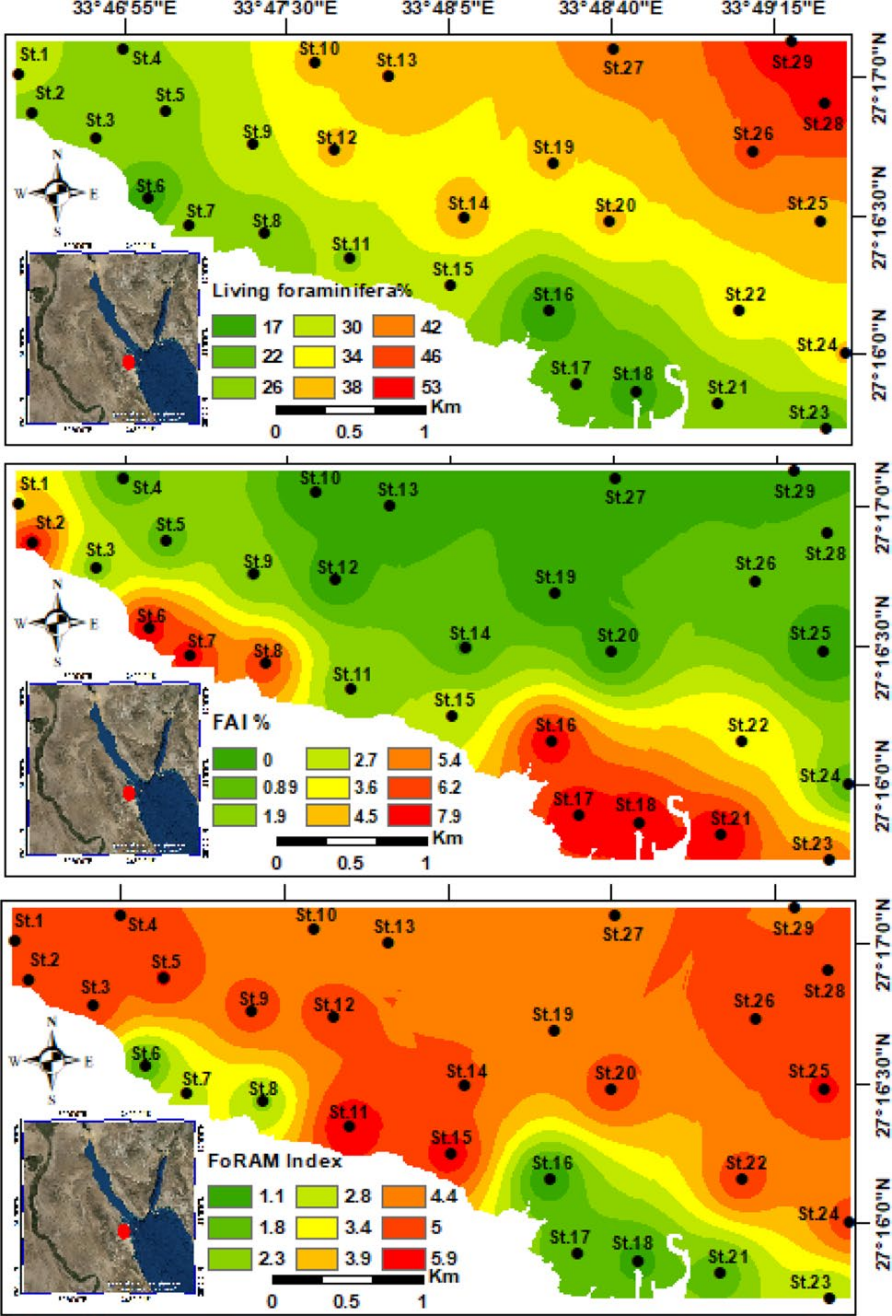
Distribution maps for the relative abundance of the living benthic foraminiferal organisms, foraminiferal abnormality index, and FoRAM index in the Hurghada site

Overall, the living benthic benthic foraminiferal individuals showed low percentages along the coastal and nearshore stations (i.e., St.6, St.16, St.17, St.18, and St.21) (Fig. 7). Toward the open marine, the living percentages have been leaped until it was reached the maximum at St.29 (53%) (Fig. 7). The northwestern and southeastern sectors had the lowest living percentages in the area of study, which ranged from 17 to 26%.

#### Benthic foraminiferal biotic indices

The foraminiferal abnormality index (FAI) discriminated two main groups of stations based on the deformation percentage. Group (1) comprises stations along nearshore areas of the northwestern and southeastern sectors (e.g., St.2, St.6, St.7, St.8, St.16, St.17, St.18, St.21, and St.23), and they are valued high FAI (5.4–7.9%). Group (2) includes stations located offshore as well as the central nearshore stations (e.g., St.3, St.4, St.5, St.9, St.10, St.11, St.14, St.15, St.22, St.24, and St.28). They are valued from low to moderate FAI% and ranging from 0 to 4.5% (Fig. 7). The benthic foraminifera in the offshore stations and central sector exhibited scarce/absent morphological deformations, clarifying the low ecological stress on these stations.

The foraminifera in reef assessment and monitoring index categorized the studied stations into three groups, based on the FoRAM index values. The stations located away from the nearshore and their faunal content inhabited the open marine, as well as the central part are higher than 4 (Fig. 7). The second group occupied the nearshore stations at the southeastern and northwestern areas and valued from 2 to 4. The third group encompasses six stations (St.6, St.8, St.16, St.17, St.18, and St.21) with values lower than 2 (Fig. 7).

**Fig. 8:**
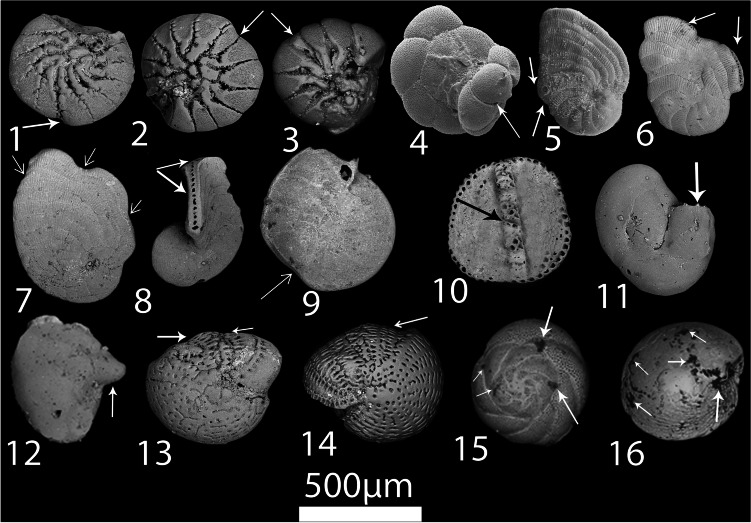
SEM micrographs of the abnormal species; 1–3: reduction in the chamber sizes of *Ammonia beccarii*, 4: abnormal growth of *Ammonia tepida* test, 5: abnormal growth and aberrant chambers of the last whorl *Peneroplis planatus*, 6–8: *P. planatus*; Siamese twin, aperture branching, and chamber reduction, 9: bifurcating the margin for *O. discoidalis*, 10: wrong direction of coiling for *A. hemprichii*, 11–12: aberrant chamber of *Quinqueloculina* cf. *seminulum*, 13–14: reductions in sizes of the last chambers of *Elphidium striatopunctatum*, 15: black oily spots of different sizes on the external surface of *Rosalina bradyi*, 16: black oily spots on the external surface of *Amphistegina lobifera*

Ten species showed forms of abnormalities in their tests. *A. beccarii* has the highest portion of deformed specimens (21.2%), pursued by *A. hemprichii* (17.5%), *E. striatopunctatum* (15.2%), *P. planatus* (14.4%), *C. hemprichii* (7.3%), *O. discoidalis* (6.5%), *N. calcar* (6.5%), *Quinqueloculina* sp. (3.5%), *A. tepida* (3.8%), and *Spiroloculina* sp. (2.6%). These foraminiferal species exhibited malformations at the Hurghada site, comprising abnormalities and deformations in their growth (Fig. 8). Some species, such as *A. beccarii*, possess multiple deformity forms (overdevelopment of the terminal chamber and lessening of chamber size), and *P. planatus* has Siamese (conjoined) twins with double apertures and branching with double apertures. *R. bradyi* and *A. lobifera* displayed oily pigments on their test structures at stations overcrowded by ships (Fig. 8).

#### Diversity indices of benthic foraminifera

**Fig. 9:**
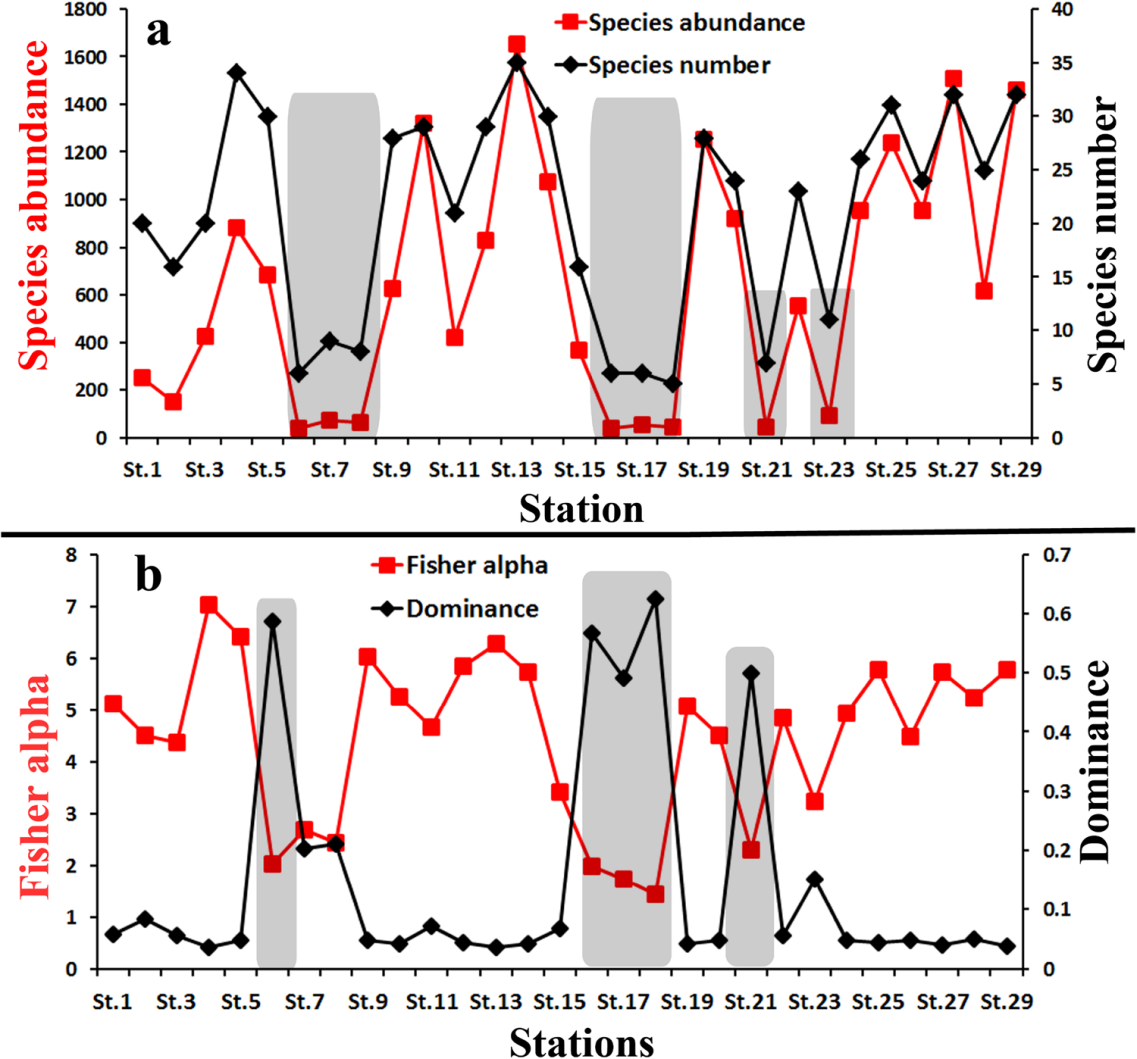
Diversity indices for the studied samples at the Hurghada site: **a** species number and species abundance; **b** Fisher alpha and dominance

The benthic foraminiferal density (FD) in the Hurghada site is high, ranging from 1666 individuals at St.13 to 70 individuals at Sts.18, 17, and 8. (Fig. 9a). The number and abundance of species show two similar patterns (Fig. 9a). The species abundance shows extremely low values in the coastal nearshore stations, especially the northwestern and southeastern sectors. These stations are St.6, St.7, St.8, St.16, St.17, St.18, St.21, and St.23 (Fig. 9a). The station encompassing the lowest species abundance is St.6 (37), followed by St.16 (39). The highest species abundance is found at St.13 (1653) and St.27 (1510). Moreover, the species richness exhibited an extremely low diversity in the nearshore coastal stations. Station (St.18) has the lowest number of species (5), followed by St.6, St.16, and St.17 (6). Conversely, St.13 has the highest species number (34), followed by St.4 (33), St.27 (32), and St.29 (32).

Regarding the diversity indices, St.18 is characterized by the lowest species diversity, as expressed by the extremely low Fisher alpha index value (1.4). In contrast, the dominance index (0.6) is the highest among all stations (Fig. 9b). Five species dominate this station: *A. tepida*,* R. bradyi*,* E. striatopunctatum*,* P. planatus*, and incredibly small numbers of *A. beccarii*. Following St.18 are the stations St.17, St.16, St.6, and St.21, which exhibited Fisher alpha values 1.7, 2, 2, and 2.3, respectively, while the dominance indexes are 0.5, 0.6, 0.6, and 0.5, respectively. These stations are highly dominated by *A. tepida*,* E. striatopunctatum*,* Q. seminulum*,* R. bradyi*, and* P. planatus*. Aside, the benthic foraminifers at St.4, St.5, S.13, and St.9 have the highest diversity indices and the lowest dominance values. The species richness is approximately five-fold higher at the offshore stations (St.26, St.13, St.27, St.29) than at the coastal nearshore stations. The diversity increases with sea-level deepening and pollution level lessening (Holt and Miller 2010).

### Statistical analyses of benthic foraminifera and environmental variables

#### Hierarchical cluster analyses (HCA)

**Fig. 10:**
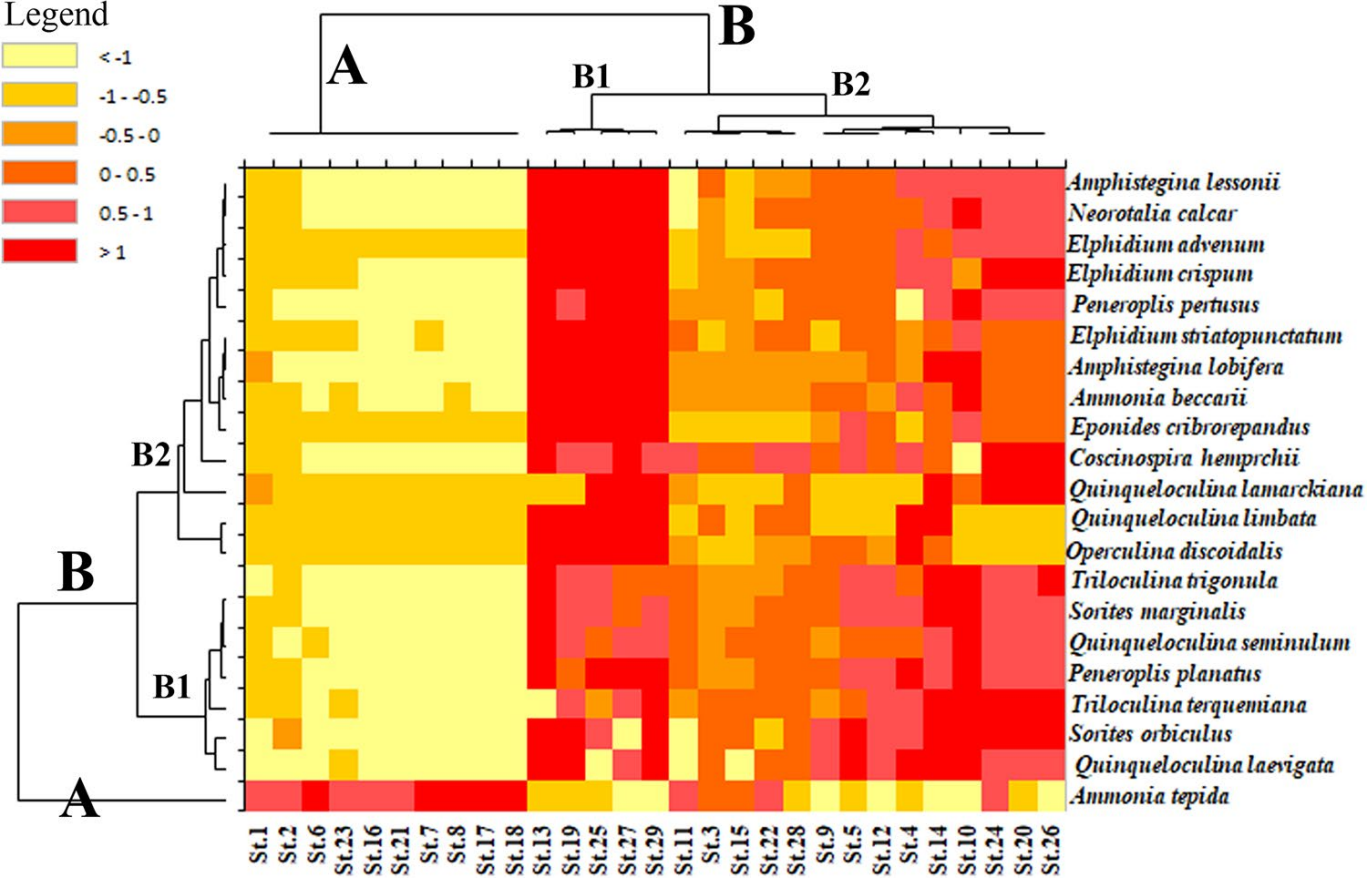
Heatmap cluster analyses via Q and R-modes based on the higher than 3% of the benthic foraminiferal taxa of the 29 variables (stations), using Ward’s method

HCA identified diverse clusters that explain different environmental biotopes. To explore similarities between stations, the Q-mode cluster analysis had been utilized. The samples categorized into two main clusters, A and B (cluster B is further divided into B1 and B2) (Fig. 10). R-mode CA was also constructed to identify the faunal assemblages and their distribution.

Cluster A is classified by the occurrences of substantial pollution characteristics. It is mainly differentiated by stations with high heavy metal contents (i.e., Pb, Zn, Ni, Cr, Cd, Mn, and As) and faunal assemblage. Furthermore, cluster A has high TOM%, fine-grained sediments, turbid water, low foraminiferal abundance, and species diversity. Stations of cluster A are St.1, St.2, St.6, St.7, St.8, St.16, St.17, St.18, St.21, and St.23 (Fig. 10). The extremely high percentages of *A. tepida* are characteristic of these stations, which are located in proximity to toxicant sources (Fig. 10).

Cluster B includes B1 and B2. Subcluster B1 encompasses St.13, St.19, St.25, St.27, and St.29, which are located away from the coastal shoreline (Fig. 10). These stations have the least heavy metal concentrations with high CaCO_3_%, TOM%, and mud contents. This subcluster is dominated by *P. planatus*,* P. pertusus*, *A. lobifera*,* A. lessonii*, *E. advenum*, *E. crispum*, *E. striatopunctatum*,* C. hemprichii*,* E. criprorepandus*, *O. discoidalis,* and* Q. limbata* (Fig. 10).

Subcluster B2 includes stations St.4, St.5, St.9, St.10, St.12, St.14, St.20, St.22, St.24, St.26, and St.28 with low pollution levels as determined from the heavy metal concentrations and benthic foraminiferal distribution (Fig. 10). These locations represent low pollution levels compared to clean and offshore stations. This subcluster is dominated by *Q. laevigata*,* T. terquemiana*,* T. trigonula*,* Q. seminulum*, *S. marginalis*, and *S. orbiculus* (Fig. 10).

#### Redundancy analysis (RDA)

**Fig. 11:**
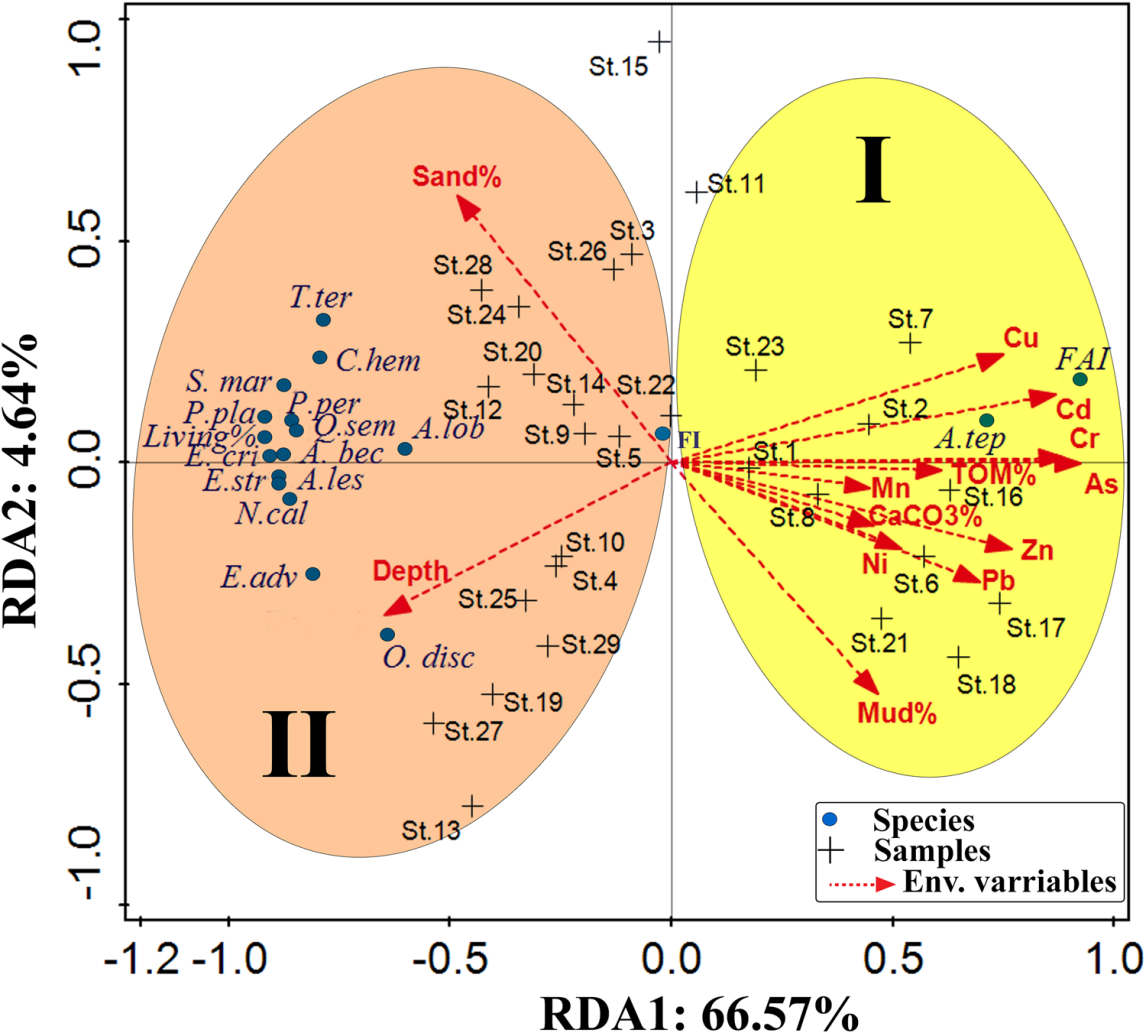
Redundancy triplot analysis shows the relationship of the first two axes of the RDA between the foraminiferal assemblages, environmental factors, and stations

The first two RDA axes account for 66.57 and 4.64% of the total variance. Since they explain a minor percentage of the total variations, higher axes were omitted. Axis 1 classifies stations into two groups depending on their environmental characteristics (Fig. 11). Group I occupy the positive area of axis 1. It consists of the most polluted stations of the Hurghada site (i.e., St.8 and St.17), and the observed tolerant species is *A. tepida* (Fig. 11). Group I include locations characterized by exceedingly high heavy metal concentrations, TOM%, mud content, and CaCO_3_%. Moreover, the highest percentage of the FAI was found in this group (Fig. 11). This group shows a positive correlation between stations and Mud%, Cd, Cr, Mn, As, Zn, Pb, Ni, Cu, TOM%, and carbonate%.

Group II is located on the negative side of the triplot chart. It consists of the least polluted or non-polluted stations apart from the tourist villages, hotels, and pollution sources. These stations have low heavy metal concentrations (e.g., St. 3, St. 4, St. 5, St.9, St.10, St.12, St.15, St.22, and St.24) (Fig. 11). The group exhibits positive correlation with the sand% and water depth, whereas negatively correlated to all the other heavy metals and environmental variables. This group is characterized by remarkably high percentages of sensitive taxa, including *E. advenum*, *S. marginalis*, *Q. seminulum, E. striatopunctatum*, *P. pertusus*, *A. beccarii, E. crispum*, *Q. laevigata*,* T. terquemiana*,* P. planatus*,* C. hemprichii*,* A. lobifera*,* N. calcar*,* O. discoidalis*, and* A. lessonii.* This group is congruent with the density of living foraminiferal species (Fig. 11). The sand content and depth are considered as the main controlling factors where they influence the distributions of foraminiferal species in this group.

## Discussion

### Heavy metals risks, sources, and assessment

The benthic foraminiferal abundance has been linked to heavy metal pollution in lots of studies (e.g., Frontalini et al. 2011; El-Kahawy et al. 2018; Price et al. 2019; El Kateb et al. 2020; Li et al. 2021; Balachandar et al. 2023). They deduced that a rise in the concentration of certain metals (e.g., Cu, Cd, Pb, and Zn), generally leads to a decline in the foraminiferal population (species richness and abundance). Benthic foraminifera in heavily polluted areas may move elsewhere, become vanished and/or morphologically deformed. Notably, the coastal and nearshore southeastern and northwestern stations have substantially high heavy metals than of the nearshore of the central stations and offshore ones (Table 2; Figs. 3 and 4). Their contributions are also much more than those of background values of Hanna (1992), Attia and Ghrefat (2013), and Nour et al. (2018), who all focused on the Hurghada area. This indicates that there is a recent and localized rise in the concentrations of the analyzed heavy metals in the shallow marine sediment of the Red Sea. A potentially risk consequence is posed by the very high concentration of lead (Pb), cadmium (Cd), and nickel (Ni) in the nearshore stations, which is much greater than that of the typical shallow sea sediment. In addition, the presence of high levels of these metals may serve as a threatening signal for possible toxicity in the Hurghada district.

On the other hand, the calculated geochemical indices displayed compatible behavior with the highly hazardous stations at the Hurghada area, where toxic metals have been discharged. The enrichment factor, geo-accumulation index, and contamination factor exhibited higher values of Pb, Cd, and Ni (Table 4), which most probably due to the anthropogenic activities at the area of study. On the other hand, the other heavy metals are less than 1, that is clarifying their natural sources. The PLI discriminated the Hurghada stations into polluted and non-polluted ones by heavy metals contaminants. The highest PLI values were calculated from northwestern and southeastern stations, where they are closer to the residential buildings, shipyard, and other human activities. This clarifies the reason why St.17 is the highest PLI (4.54), as well as the other nearshore stations shown bold in the Table 5a. Additionally, the SQGs displayed high heavy metals content for the mean of most analyzed metals than the LEL of Persuad et al. (1993), and lower than SEL. Interestingly, the nearshore stations (i.e., St.7, St.16, St.17, St.18, St.21, and St.23) are higher than the LEL values for all of the measured heavy metals, implying an adverse effects by heavy metals on aquatic organisms like benthic foraminifera or coral reefs. This is obviously from the FoRAM index values of stations St.6, St.16, St.17, St.18, and St.21 that showed very low values (< 2) indicating stressful conditions hindering the reefs growth. Additionally, stations such as St.7, St.8, and St.23 are valued between 2 to 4 indicating that the environment is marginal for reef growth and unsuitable for recovery. The field data shows that shipping, urban sewage, phosphate mining, and, to a lesser degree, oil drilling in the Red Sea region to the north of the Hurghada Bay are the essential contributors to the pollution there.

The heavy metals risk on human health throughout non-carcinogenic and carcinogenic attempts has been assessed and exhibited interesting values. The non-carcinogenic risk for children and adults is less than 1 indicating no experienced of any health risk due exposure to non-carcinogenic metals. This result is coincided with Nour et al. (2022), which reported that both sands and soil of the Hurghada Bay are safe for both children and adults. Aside, the carcinogenic risk showed very high Pb, As, and Cr contents for the adult and children either through ingestion or dermal contact, and for LCR. Nour et al. (2022) claimed that the Hurghada area is safe, and their heavy metals lies within the range of the permissible limit. However, their conclusion was based on one sample only collected from the Hurghada beach, which was not representative and insufficient to evaluate an overcrowded city by shipping activities and buildings. Consequently, our data displayed higher carcinogenic hazard effects on the adults by Pb, As, Cr, Cd, and Ni, while children by Pb, As, Cr, and Ni respectively. The Pb represents the highest carcinogenic value for the adult and children, which may cause immune imbalance, intellectual disability, skeletal delay, vitamin D deficiency, and hearing loss due to high exposure (USEPA 2011; Luo et al. 2012).

### Foraminifers as bioindicators of pollution

In chiefly, two groups of benthic foraminifera were classified based on their distribution behavior in response to the anthropogenic activities in the Hurghada site.

## Group I

Group I arise where sediments are characterized by enriched heavy metal concentrations, TOM%, mud content, high-water temperature, low salinity, and pH. The group is categorized by a high proportion of *A. tepida* in the polluted stations St.16, St.21, St.17, St.18, St.6, St.8, and St.23. According to Alve (1991), opportunistic and resistant species could thrive in polluted ecosystems. In contrast, Yanko et al. (1999) proposed the idea of species responsiveness to pollutants through their disappearance. *A. tepida* has been reported as a highly abundant indicator for wastewater (Seiglie 1971), industrial wastes (Seiglie 1975), chemical and agricultural wastes (Setty 1976), and heavy metals (i.e., Nagy and Alve 1987; Alve 1991; Samir and El-Din 2001; Frontalini and Coccioni 2008). According to Vilela et al. (2004), *A. tepida* was highly populated in the stations contaminated by heavy metals of Guanabara Bay, suggesting that it is an opportunistic species. These results coincide with the findings of our investigation, which shows that *A. tepida* is the dominating species at the intensively contaminated stations. Furthermore, living benthic foraminifers were scarce at the highly polluted stations (e.g., St.6, St.8, St.16, St.17, St.18, St.21, and St.23), where it is confirming resistance to pollution as an opportunistic species. On other hand, Samir et al. (2003) reported it as a prolific species in calm environments whose bottom sediments are muddy or sandy, while Debenay et al. (2006) have documented its abundance in turbid estuary settings where it favors reduced salinities (Walton and Sloan 1990). The present study showed that* A. tepida* populates the low salinities and organic-rich fine-grained substrate of highly turbid stations (i.e., St.17 and St.21). Moreover, *Ammonia* spp. proved their sensitivity to varying levels of environmental degradation. Noteworthy, *A. tepida* has been identified as a pollution-tolerant taxon, whereas *A. beccarii* is a sensitive species. This is supported by their affinities toward polluted and non-polluted stations, respectively, which is well-matched with Poag (1978) and Samir (2000).

## Group II

Group II consists of benthic foraminiferal assemblages comprising *P. planatus*,* C. hemprichii*, *Q. laevigata*,* Q. seminulum*, *T. terquemiana*, *N. calcar*, *S. marginalis*,* P. pertusus*,* A. lobifera*,* A. lessonii*,* E. advenum*,* E. crispum*,* E. striatopunctatum*, *A. beccarii*, and* O. discoidalis.* This group assemblage displays its highest occurrences at stations characterized by low pollution levels and high coarse sediments (sand fraction). The main controlling factor for the abundance of this foraminiferal assemblage is the sand content, where it was displayed a strong correlation coefficient. Also, HCA and RDA further confirmed the sensitivity of some of these genera (e.g., *Quinqueloculina*, *Peneroplis*, and *Coscinospira*) to stressed conditions. Rao and Rao (1979) and Samir and El-Din (2001) had deemed that miliolids are less resistant to pollutants, which is consistent with our results. Although the pollution sensitivity of *Quinqueloculina* spp., other studies recorded different *Quinqueloculina* species as pollution indicators. Accordingly, Romano et al. (2009) recognized *Quinqueloculina parvula* as a pollution-tolerant species. Also, *Elphidium excavatum* shows tolerance to most contaminants (Schafer et al. 1991). In the present work, the miliolids and *Elphidium* spp., have been observed in high abundance at stations off low pollution levels and low abundance with high pollution levels. Accordingly, this may be clarifying the deformations and morphological abnormalities of the miliolids and *E. striatopunctatum* in the polluted stations. Also, these sensitive taxa might disappear from the contaminated area as an alternative strategy reflecting the impacts of the TOM% and pollution by heavy metals enrichments.

### Diversity indices and test abnormalities

Using species diversity, the effect of environmental stress on benthic foraminiferal communities could also be assessed. Consequently, polluted environments have limited species diversity (Samir and El-Din 2001). In Chaleur Bay, eastern Canada, Schafer (1973) found that species diversity diminishes closer to effluent sources. It has been found that foraminiferal species diversity and density increases with distance from a pollution source in the eastern US Chesapeake Bay (Bates and Spencer 1979). Schafer et al. (1991) also found that under stressed habitats, foraminiferal diversity and density were significantly reduced. Alve (1991) and Burone et al. (2006) showed a distinct foraminiferal response, with intermediate levels of diversity coupled with low faunal density at the most contaminated spots. On the other hand, Alve and Olsgrad (1999) deduced a statistically significant negative correlation between the density of foraminifers and rising Cu content in sediments. Marine benthic ecosystems were devastated by heavy metal pollution, leading to the near-complete eradication of forams and other organisms (Samir 2000; Ferraro et al. 2006). Murray (1973) and Pearson and Rosenberg (1976) assert that rising pollution results in a poor community dominated by a few opportunistic species. These results are well-matched with our study, confirming that pollution by heavy metals leads to low diversity foraminiferal communities with resistant taxa domination, especially at the heavily contaminated stations St.6 and St.17. Moreover, strong negative correlation coefficients are obtained between the species diversity and all the analyzed heavy metals, which clarifies the negative impact of heavy metals on the species diversity (Table 3). Other environmental factors also influence the diversity of foraminifera. The low diversity, particularly in highly contaminated stations such as St.6, St.7, St.8, St.16, St.17, St.18, St.21, and St.23, may be explained due to the high TOM%. Adversely, according to Loubere (1997), foraminiferal diversity is rising in well-oxygenated sediments; however, organic matter enrichments influenced the oxygen availability around oxygenated stations, causing a low species diversity (i.e., St. 17 and St.18). The correlation analysis found a strong negative correlation coefficient (− 0.7) between the organic matter content and the foraminiferal diversity (Table 3).

## Conclusion

Based on 29 sediment samples collected from the Hurghada Bay, the environmental status has been evaluated using benthic foraminiferal and geochemistry of sediments. The present study yielded 34 benthic foraminiferal species belonging to 21 genera, and three suborders. Some of the recorded benthic foraminifers displayed deformations in their structures due to extreme environmental stress. The chemical analyses of the heavy metals revealed that the higher concentrations were distributed along the nearshore stations where there are huge quantities of sewage and industrial and fishing activities. Furthermore, the living foraminifers in the nearshore stations are low, especially the polluted ones, whereas the dead foraminifers are extremely high. Geochemically, the EF, CF, PLI, and *I*_geo_ were used to evaluate the contamination of the bottom sediments. Moreover, the carcinogenic and non-carcinogenic heavy metals risks showed significant impacts on adults and children. Hurghada Bay suffered from anthropogenic influences as observed during the field sampling, e.g., tourist resorts, dredging, and land reclamation. For that, the environmental impact assessments are an urgent and vital necessity in such environments to monitor and evaluate the present ecosystem and keep the coral reefs maintained along the Red Sea coast.

## Supplementary Information

Below is the link to the electronic supplementary material.

Supplementary file1 (TIF 873 KB)

Supplementary file2 (TIF 216 KB)

## Data Availability

The data described in this study can be obtained from the corresponding author upon request.

## Abbreviations

A. tep: *Ammonia tepida*
A. bec: *Ammonia beccarii*
A. lob: *Amphestigina lobifera*
A. les: *Amphestigina lessonii*
N. cal: *Neorotalia calcar*
O. disc: *Operculina discoidalis*
S. mar: *Sorites marginalis*
E. str: *Elphidium striatopunctatum*
E. cri: *Elphidium crispum*
E. adv: *Elphidium advenum*
P. pla: *Peneroplis planatus*
P. per: *Peneroplis pertusus*
Q. sem: *Quinqueloculina seminulum*
T. ter: *Triloculina terquemiana*
C. hem: *Coscinospira* Hemprichii
