# Supplementary figures and images for: Benthic foraminifera as bioindicators for the heavy metals in the severely polluted Hurghada Bay, Red Sea coast, Egypt
Source: Environ Sci Pollut Res Int. 2023 May 6;30(27):70437–57. doi: 10.1007/s11356-023-27242-4 (PMC10239395; doi:10.1007/s11356-023-27242-4)

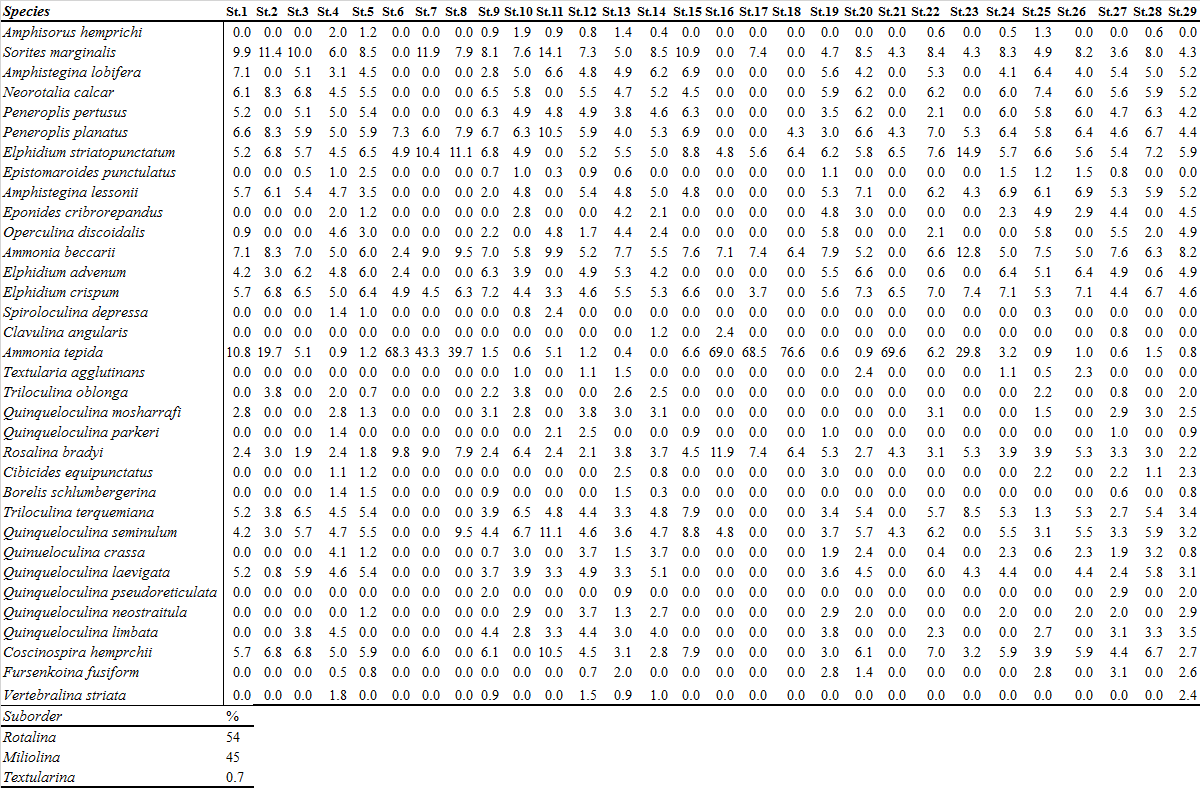

Supplement: Supplementary file 1 — Supplementary file1 (TIF 873 KB) [file 11356_2023_27242_MOESM1_ESM.tif]

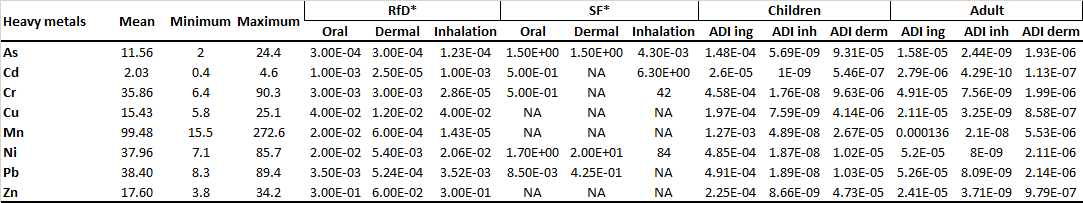

Supplement: Supplementary file 2 — Supplementary file2 (TIF 216 KB) [file 11356_2023_27242_MOESM2_ESM.tif]
